# Effects of Graphene Oxide Nanosheets in Freshwater Biofilms

**DOI:** 10.3390/molecules28124577

**Published:** 2023-06-06

**Authors:** Diana Matos, Salomé F. P. Almeida, Paula A. A. P. Marques, Sofia Pinto, Etelvina Figueira

**Affiliations:** 1 Department of Biology, University of Aveiro, 3810-193 Aveiro, Portugal; dianarmatos@ua.pt (D.M.); salmeida@ua.pt (S.F.P.A.); sofiamaia.pinto@ua.pt (S.P.); 2 Centre for Environmental and Marine Studies (CESAM), University of Aveiro, 3810-193 Aveiro, Portugal; 3 GeoBioTec, GeoBioSciences, GeoTechnologies and GeoEngineering Research Centre, University of Aveiro, 3810-193 Aveiro, Portugal; 4 Department of Mechanics, University of Aveiro, 3810-193 Aveiro, Portugal; paulam@ua.pt; 5 TEMA, Centre for Mechanical Technology and Automation, Department of Mechanical Engineering, University of Aveiro, 3810-193 Aveiro, Portugal

**Keywords:** graphene oxide, freshwater biofilms, shading, photosynthetic pigments, oxidative damage, antioxidant activity

## Abstract

Graphene oxide (GO) properties make it a promising material for graphene-based applications in areas such as biomedicine, agriculture, and the environment. Thus, its production is expected to increase, reaching hundreds of tons every year. One GO final destination is freshwater bodies, possibly affecting the communities of these systems. To clarify the effect that GO may impose in freshwater communities, a fluvial biofilm scraped from submerged river stones was exposed to a range (0.1 to 20 mg/L) of GO concentrations during 96 h. With this approach, we hypothesized that GO can: (1) cause mechanical damage and morphological changes in cell biofilms; (2) interfere with the absorption of light by biofilms; (3) and generate oxidative stress, causing oxidative damage and inducing biochemical and physiological alterations. Our results showed that GO did not inflict mechanical damage. Instead, a positive effect is proposed, linked to the ability of GO to bind cations and increase the micronutrient availability to biofilms. High concentrations of GO increased photosynthetic pigment (chlorophyll a, b, and c, and carotenoids) content as a strategy to capture the available light more effectively as a response to the shading effect. A significant increase in the enzymatic (SOD and GSTs activity) and low molecular weight (lipids and carotenoids) antioxidant response was observed, that efficiently reduced oxidative stress effects, reducing the level of peroxidation, and preserving membrane integrity. Being complex entities, biofilms are more similar to environmental communities and may provide more accurate information to evaluate the impact of GO in aquatic systems.

## 1. Introduction

Carbon nanoparticles, with a dimension of less than 100 nm have been increasingly used due to their unique properties [1].

One of the best-known carbon nanoparticles is graphene. Graphene is a two-dimensional sheet of carbon atoms arranged in a hexagonal lattice [2]. Graphene can be oxidized, forming graphene oxide (GO), a form of graphene that has oxygen-containing functional groups on its surface such as epoxide, hydroxyl, carboxyl, and ketone group, increasing GO interaction with aqueous solutions [3,4]. GO has good electrical conductivity, which makes it useful in electronics and energy storage applications [5]. GO is highly hydrophilic, it can easily disperse in water, making it useful in applications such as coatings and composites. GO has excellent mechanical properties, including high tensile strength and flexibility, making it useful in applications that require strong, flexible materials [6]. The GO small size, the ability to diffuse through membranes, and the biocompatibility to create scaffolds makes it a nanoparticle of election for investigation to different biomedical applications such as drug delivery, biosensors, and tissue engineering [7,8]. The large surface area provided by the two-dimensional structure, high reactivity, and ability to adsorb a wide range of pollutants, makes GO suitable for removal of heavy metals, organic contaminants, water purification, and desalination [4,9,10,11]. The high surface area, reactivity, and stability are also important for GO application in agriculture, such as to improve soil water retention and nutrient availability, promote plant growth by photosynthesis enhancement and nutrient uptake by plants, and targeted delivery of agrochemicals to crops, reducing the amount of chemicals needed and minimizing environmental impact [12,13]. Due to GO properties, the real and potential applications [14], its production has increased, reaching hundreds of tons every year [15], and may lead in the future to scenarios of increased GO in the environment. Once released into the environment, GO can enter streams and rivers through industrial and municipal wastewater discharges, and runoff from agricultural and urban areas [16]. Currently it is difficult to detect trace concentrations of carbon nanoparticles (CNPs) in the environment [17]. Environmentally relevant concentrations (ERCs) of CNP in water, based on a stochastic/probabilistic material-flow computer model, are in the μg/L or ng/L range [18] while the predicted environmental concentrations (PECs) were projected to approximately 0.001–1000 μg/L [19,20].

Since freshwater systems harbor communities sensitive to disturbance, the presence of GO nanoparticles, even at low concentrations, can induce changes in these communities. Biofilms are important communities of freshwater systems. These biofilms are assemblages of microorganisms including bacteria, archaea, fungi, algae, protozoa, and viruses embedded in a matrix of extracellular polymeric substances, produced by the microorganisms therein, usually diatoms are the dominant group [21]. Bacteria and fungi are also important contributors of biofilms, as they play critical roles in decomposing and recycling organic matter within the biofilm communities [22]. These communities attach and grow on submerged surfaces in aquatic ecosystems, such as rocks, plants, and sediments. Biofilms can be an important source of primary production in aquatic ecosystems, providing the base of the food web and supporting the growth of higher trophic levels. Biofilms also play an important role in nutrient cycling by taking up nutrients such as nitrogen and phosphorus, but also pollutants, thus improving water quality. All these ecological functions make freshwater biofilms an important component of aquatic ecosystems, contributing to the functioning and productivity of freshwater rivers and streams [21]. Understanding the factors influencing biofilm communities can provide important insights into the health and resilience of aquatic ecosystems. Although the impact of GO on rivers and streams is poorly investigated, GO was reported to have several effects on algae cultures and on biofilms, including both beneficial and detrimental. Some studies have reported that low GO concentrations did not significantly affect the growth and photosynthetic activity of algal cultures and biofilms, and even promoted growth. GO had a positive effect on the growth and pigment content of a green algae, *Picochlorum* sp. at low concentrations (0.5 mg/L), but a negative effect was observed at 5 mg/L [23,24]. No adverse effects were observed on the growth of the cyanobacteria Microcystis aeruginosa exposed to 5 mg/L GO, but a significant decrease was observed at concentrations above 15 mg/L [24,25]. In contrast, cell division of *Chlorella vulgaris*, another green algae, was inhibited after exposure for 96 h to all the concentrations tested (0.01, 0.1, 1, and 10 mg/L) [24,26]. GO concentrations between 0.1 and 10 mg/L did not influence the growth of the diatom *Nitzschia palea* growth, but a significant decrease in growth and photosynthetic activity was observed at 50 mg/L [27]. Authors related the promotional effect by GO acting as a source of carbon and other nutrients that are beneficial for algal growth. Indeed, the zeta potential of GO is negative [28], and the negative surface can interact with cations, such as Mg, Fe, Ca, K, and Na [8]. On the other hand, the negative effect of GO on algae cultures was linked to the ability of GO to generate reactive oxygen species (ROS) that can cause oxidative damage, shading effects, and mechanical damage [14,24,27,29]. This can lead to reduced growth and even death. The reported inhibition of high GO concentrations on algae photosynthesis, which can further affect their growth and survival, was associated to the ability of GO to block or interfere with the absorption of light [14,24,27,29]. Studies have also reported that exposure to GO can lead to changes in the morphology of green algae *Chlorella vulgaris* and *Chlorella pyrenoidosa*, such as changes in cell shape and size, which may be indicative of cellular damage and stress [26,30]. Thus, contradicting effects of GO have been reported, and may be linked to factors such as concentration and exposure time and point to the need for further research to clarify the potential environmental risks of GO in the aquatic environment and to ensure that GO is used in a safe and sustainable manner.

To clarify the effect that GO may impose in freshwater biofilms, lab experiments were carried out exposing a fluvial biofilm community scraped from submerged river stones to a range (0.1 to 20 mg/L) of GO concentrations during 96 h. With this approach, we hypothesized that: (1) GO mechanical damage and morphological changes in cell biofilms; (2) GO interferes with the absorption of light by biofilms; (3) GO generates oxidative stress, causing oxidative damage and inducing biochemical and physiological alterations.

## 2. Results

### 2.1. Morphology and Elements Abundance Analysis by Scanning Electron Microscopy (SEM-EDX)

**Figure 1:**
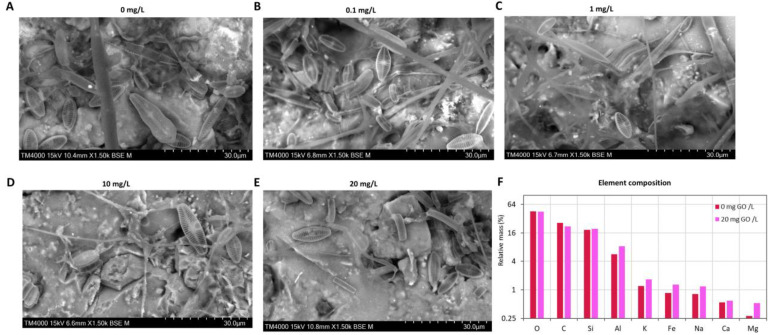
SEM images of biofilms exposed to different concentrations of GO. (**A**) Biofilm not exposed to GO control. (**B**) Biofilm exposed to 0.1 mg/L of GO. (**C**) Biofilm exposed to 1 mg/L of GO. (**D**) Biofilm exposed to 10 mg/L of GO. (**E**) Biofilm exposed to 20 mg/L of GO. (**F**) Element composition of biofilm at control and 20 mg/L of GO obtained by EDX.

The SEM images of tiles (4 × 5 cm) with the biofilms exposed to different GO concentrations revealed no teratologies or alterations in size of the organisms visualized, predominantly diatom species (Figure 1A–E).

Energy-dispersive X-ray spectroscopy (EDX) allowed to obtain the elemental composition of the biofilms exposed to 0 and 20 mg/L of GO. Oxygen (O), carbon (C), and silica (Si) are the most abundant elements (Figure 1F). Aluminum (Al), potassium (K), iron (Fe), sodium (Na), calcium (Ca), and magnesium (Mg) were also determined in the biofilm samples (Figure 1F).

Oxygen, carbon, silica, and calcium values were similar in biofilms not exposed to GO (control) and to the highest GO concentration (20 mg/L). Nevertheless Al, K, Fe, and Na all increased between 40.0% and 51% at 20 mg/L of GO compared with the control (Figure 1F). The highest increase was observed for Mg. At 20 mg/L GO Mg content increased 89%.

### 2.2. Photosynthetic Pigments

**Figure 2:**
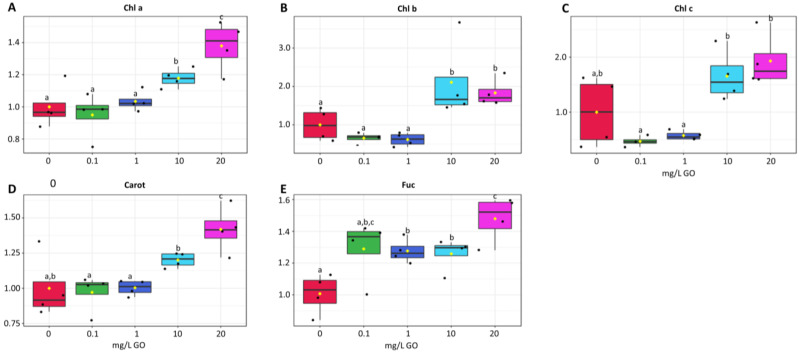
Photosynthetic pigment changes in biofilms exposed to GO (0 mg/L, 0.1 mg/L, 1 mg/L, 10 mg/L, and 20 mg/L). Results are expressed as variation relative to control (0 mg/L GO). (**A**) chlorophyll a (Chl a). (**B**) chlorophyll b (Chl b). (**C**) chlorophyll c (Chl c). (**D**) carotenoids (Carot). (**E**) fucoxanthin (Fuc). Values are means of three replicates + standard deviation. Significant differences compared with non-exposed biofilm (control) were marked with lowercase letters (*p* < 0.05). For means, standard errors, and statistical difference see Supplementary Table S1.

At the lower GO concentrations (0.1 and 1 mg/L) chlorophyll a maintained similar levels to the control, yet at the highest concentrations (10 and 20 mg/L) chlorophyll a increased significantly (Figure 2A). The same trend was observed for chlorophyll b (Figure 2B) and chlorophyll c (Figure 2C).

Carotenoids content was also maintained at 0.1 and 1 mg GO/L and increased significantly at 10 and 20 mg/L (Figure 2D). Fucoxanthin, an abundant carotenoid in diatoms, increased at all GO concentrations tested compared with the control, but were only statistically significant at the three highest GO concentrations (Figure 2E). At 20 mg/L, the increase in fucoxanthin was the highest recorded, being significantly different from other conditions.

### 2.3. Cell Metabolism

**Figure 3:**
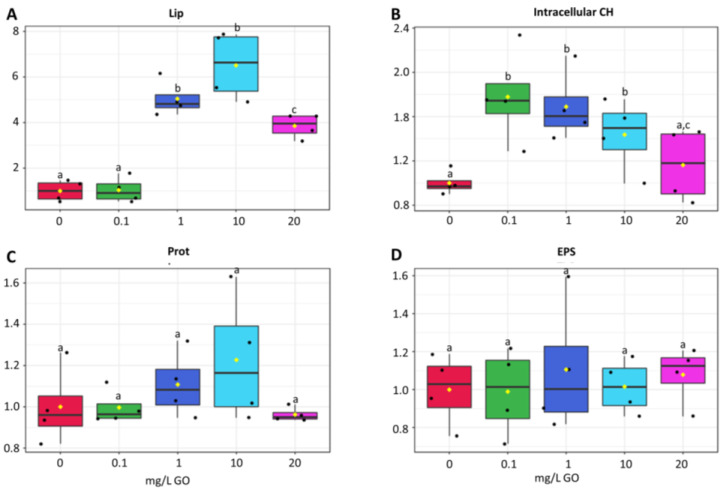
Effects of GO (0 mg/L, 0.1 mg/L, 1 mg/L, 10 mg/L, and 20 mg/L) on cellular metabolism of biofilms. Results expressed as variation relative to control (0 mg/L GO). (**A**) lipid content (Lip). (**B**) intracellular carbohydrates (Intracellular CH). (**C**) protein content (PROT). (**D**) Exopolysaccharides (EPSs). Values are means of three replicates + standard deviation. Significant differences compared with non-exposed biofilm (control) were marked with lowercase letters (*p* < 0.05). For means, standard errors, and statistical difference see Supplementary Table S1.

The lipid content was not different in biofilms exposed to the lower GO concentration (0.1 mg/L), but significantly higher levels were observed at 1, 10, and 20 mg/L of GO, with 10 mg/L showing the highest increase (700%) compared with the control (Figure 3A).

Total sugar content increased sharply at 0.1 mg GO/L, then decreased as GO concentration increased and at 20 mg GO/L, sugar content was not significantly different from the control (Figure 3B)

No significant differences were observed in soluble protein content among conditions (Figure 3C). However, increases were observed at 1 and 10 mg/L (Figure 3C).

No significant changes were observed in the exopolysaccharide (EPS) content of biofilms after exposure to any of the GO concentrations (Figure 3D).

### 2.4. Antioxidant and Biotransformation Response

**Figure 4:**
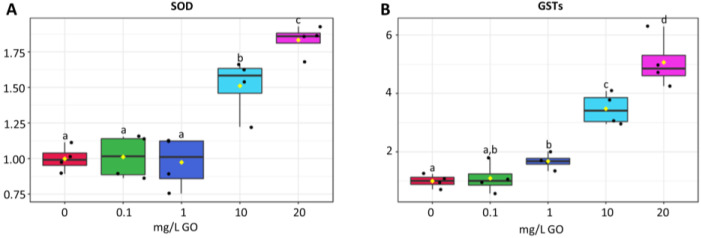
Antioxidant and biotransformation response of biofilms exposed to GO (0 mg/L, 0.1 mg/L, 1 mg/L, 10 mg/L, and 20 mg/L). Results expressed as variation relative to control (0 mg/L GO). (**A**) superoxide dismutase (SOD). (**B**) glutathione-s-transferases (GSTs). Values are means of three replicates + standard deviation. Significant differences compared with non-exposed biofilm (control) were marked with lowercase letters (*p* < 0.05). For means, standard errors, and statistical difference see Supplementary Table S1.

SOD activity remained low at 0.1 and 1 mg/L GO, increasing significantly at the two highest GO concentrations (52 and 85% for 10 and 20 mg/L, respectively) (Figure 4A).

GSTs activity at 0.1 mg/L GO remained similar to the control, but significant increases were observed at 1, 10, and 20 mg/L (Figure 4B). The highest concentration of GO evidenced the highest induction of GSTs activity (434%).

### 2.5. Cell Damage

**Figure 5:**
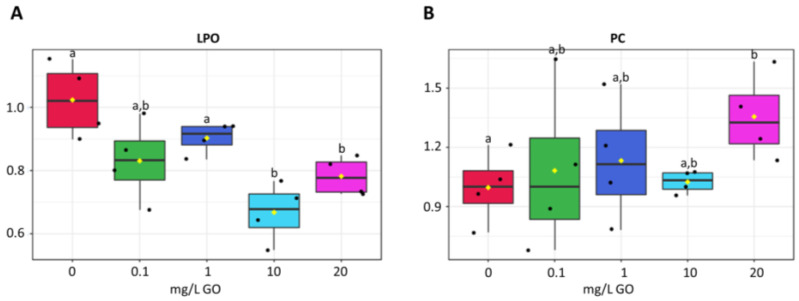
Effects of GO (0 mg/L, 0.1 mg/L, 1 mg/L, 10 mg/L and 20 mg/L) on cell damage of biofilms. Results expressed as variation relative to control (0 mg/L GO). (**A**) lipid peroxidation (LPO). (**B**) protein carbonilyation (PC). Values are means of three replicates + standard deviation. Significant differences compared with non-exposed biofilm (control) were marked with letters (*p* < 0.05). For means, standard errors, and statistical difference see Supplementary Table S1.

Lipid peroxidation decreased between 11 and 34% in all concentrations tested relative to the control, being significantly lower at 10 and 20 mg/L (Figure 5A).

Protein carbonylation levels were higher in cells exposed to all GO concentrations, yet a significant increase was only observed at 20 mg/L GO (Figure 5B).

### 2.6. PCA

**Figure 6:**
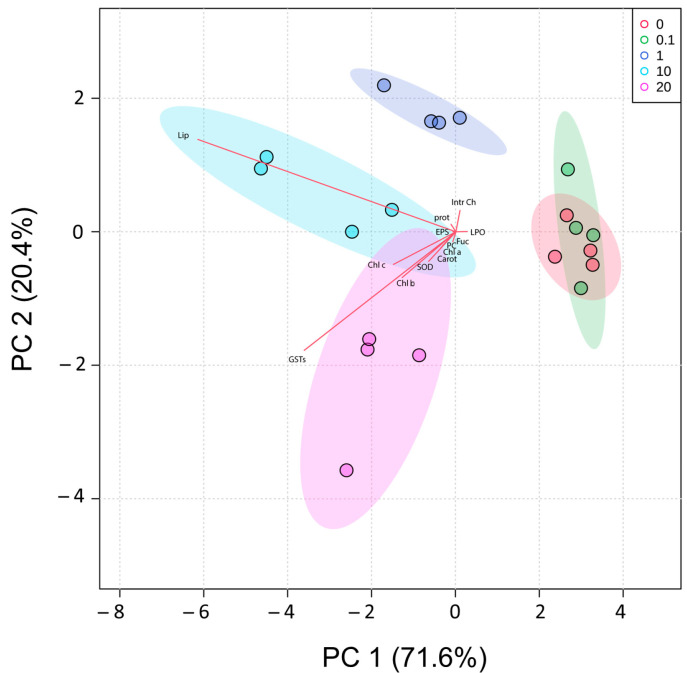
Principal Component Analysis (PCA) of biochemical changes induced by GO (0 mg/L, 0.1 mg/L, 1 mg/L, 10 mg/L, and 20 mg/L) in biofilms. Pearson correlation vectors were imposed: lipid peroxidation (LPO); protein carbonylation (PC); protein (PROT); lipids (Lip); intracellular carbohydrates (intra Ch); exopolysaccharides (EPSs); superoxide dismutase activity (SOD); glutathione S-transferases (GSTs); chlorophyll a (Chl a); chlorophyll b (Chl b); chlorophyll c (Chl c); fucoxanthin (Fuc); carotenoids (Carot).

The alterations in cellular biochemistry caused by exposure of biofilms to different concentrations of GO are represented in a Principal Components Analysis (PCA). Together, PC1 and PC2 explained 92% of the total variation obtained among conditions (Figure 6). Along PC1, the control (0) and 0.1 mg/L GO conditions were clearly separated on the positive side of the axis and the remaining conditions on the negative. PC2, explained 20.4% of total variation, separating the condition 20 mg/L in the negative side of axis 2, from 1 and 10 mg/L conditions on the positive side.

## 3. Discussion

The different applications envisaged for GO will certainly increase its use in the near future, consequently, GO will irremediably end up in water bodies, with poorly known consequences for aquatic communities. By exposing a biofilm collected in river stones to a wide range of GO concentrations, we expected to gain knowledge (elucidate) on the impact that this molecule can have on aquatic communities. Based on reported results of GO effects, mostly on monoalgal cultures [16,20,25,26], the following hypotheses were formulated: (1) GO causes mechanical damage and conformational changes in cell biofilms; (2) GO interferes with the absorption of light by biofilms; (3) GO can generate oxidative stress, causing oxidative damage and inducing biochemical and physiological alterations.

In our work we used a combination of SEM-EDX spectroscopy to perform a morphological analysis and element abundance analysis. Images obtained by SEM do not show any teratologies or changes in the size of identifiable cells in the biofilm exposed to the GO concentrations tested.

Oxygen, carbon, and silica were the most abundant elements as expected, since carbon is the backbone of most organic molecules, and the frustules of diatoms are essentially fabricated of silicon dioxide. However, in the presence of GO we do not see significant changes in these three elements. The absence of any change in silica content may suggest that frustules remain intact, which is supported by our SEM images where no mechanical damage in the frustule surface is evident. In agreement with this result, EPS content was also not changed by GO. Since EPS functions both as a chemical and a mechanical protection mechanism [31], the non-induction of EPS at any GO concentration reinforces that GO did not inflict mechanical damage in the cells from the biofilm. Results from our study are far from being consensual. Yin et al. [24] found that the cell wall of the diatom *Cyclotella* sp. was destroyed and fragmented by exposure to GO, and it was suggested that the effect was due to mechanical damage by the sharp edges of GO [24]. As we mentioned before, in our work mechanical damage is not evident, nor were mechanical protection mechanisms induced. A possible explanation can be the aggregation of GO with less ability to inflict mechanical injury [8]. During the process of aggregation, GO nanosheets have the ability to undergo shape changes, such as 1D tube-like carbon material, 2D GO planes overlapped multiple times, and 3D sphere-like particles [8]. These forms can then combine to create GO aggregates that resemble a sphere [8]. Ouyang et al. [26] mentioned that dispersed and small-sized graphene molecules are more toxic than their aggregated counterparts. A second motive for the low mechanical injury of GO can be that in biofilms cells are embedded in a polymeric matrix [32], which appears to confer protection to cells from exposure to GO, not being necessary to increase EPS.

The surface potential of GO is mainly negatively charged, which results from the ionization of the oxygen-containing functional [8], enabling this material to bind cations [7]. The ability of GO to sorb specific target ions, and the extent to which similar ions are sorbed depend on the concentration, and on the relative affinity for the binding sites on the sorbent surface [8]. As a result, the number of ions associated with GO varies, since competing ions occupy different binding sites [2]. The ability of GO to retain a range of metal cations and settle is key to biofilms, since bound nutrients become close to biofilms and solubilizing strategies of organisms in the biofilm can increase the availability of nutrients, which would otherwise remain soluble in the water column and largely inaccessible to the biofilm organisms. EDX analysis evidenced a general increase (between 40% and 89%) in the content of micronutrients (K, Fe, Na, and Mg) in the presence of 20 mg/L GO compared with the control. Li et al. [28] showed that the ability of GO to bind cations is higher for di than for monocations, which can explain the high increase (89%) in Mg content compared with other micronutrients that are monocations (40–51%). Mg is an essential micronutrient for algae, as it is a critical component of the chlorophyll molecule and is required for proper photosynthetic function and for enzyme reactions involving ATP as coenzyme [33]. Thereby, the increase in Mg can support the increase in chlorophyll observed in our study and the general increase in metabolism that the increase in soluble protein content suggests, as most of this protein fraction has catalytic activity. Moreover, the increase in Fe is also very important for the photosynthetic process, as many electron transporters in the photosystems contain iron and antioxidant enzymes, such as catalase and some SOD isoforms, also contain Fe as a cofactor. Potassium is important for cell osmoregulation and for enzyme activity, such as the activity of the enzyme pyruvate kinase, which plays a crucial role in the production of energy (ATP) during photosynthesis [34] and helps to maintain the electrical potential across plasma membrane [35].

In this way, the first hypothesis, that GO can cause mechanical damage and conformational changes in cell biofilms, must be rejected since the highest concentration of GO tested did not induce mechanical or conformational damage on cells. On the contrary, the ability of GO to bind micronutrients can have a positive effect on biofilm growth and tolerance to GO.

The interactions of GO nanosheets with water create stable suspensions that remain in the water column [36]. GO nanosheets can also interact with cations, form aggregates, and settle [8]. In both cases a shading effect is produced that decreases the light reaching the riverbed where biofilms are found. In fact, there are several studies reporting the shading effect caused by GO nanosheets [14,16,23,37]. Long et al. [38] reported that the shading effect is one of the major factors contributing to the effects induced by GO on algae. Shading causes impacts on chlorophyll content in algae, and both increases and decreases have been reported. Tang et al. [20] did not find a significant difference in chlorophyll a content between the control and 1 mg/L GO exposed *Microcystis aeruginosa* cells, while a significant reduction was observed at 10 mg/L. Ouyang et al. [26] also reported a decrease in chlorophyll a in *Chlorella vulgaris* exposed to GO nanosheets. Yin et al. [24] reported both increases and decreases in chlorophyll a content in algae exposed to GO. Yin et al. [24] explained the different susceptibility to shading caused by GO, with the ability of algae species to migrate, especially vertically (8). The migration in the water column enables to access adequate light [39] and allows algae exposed to high GO concentrations (10 mg/L) to maintain photosynthesis with similar or higher chlorophyll levels than the control [24]. On the contrary, algae species unable to remain suspended in the medium evidenced a significant reduction in chlorophyll a [24]. In our study, most of the photosynthetic pigments (chlorophylls and carotenoids) did not change at lower GO concentrations (0.1 and 1 mg/L), increasing at higher concentrations (10 and 20 mg/L). The cell migration theory used by Yin and collaborators [24] cannot explain our results since photosynthetic cells in the biofilm have limited mobility, and therefore there must be an alternative solution to overcome the decrease in light intensity reaching the biofilm surface. Since some algae can increase the content of chlorophylls and carotenoids at low light intensities [40,41] we propose that the increase in photosynthetic pigments may enhance algae ability to capture light available at lower intensities.

In this way, the second hypothesis, that GO interferes with the absorption of light by biofilms, is confirmed, inducing the synthesis of chlorophylls to counterbalance the lower light intensity that GO nanosheets intercept.

The generation of reactive oxygen species (ROS) is a common toxicity indicator for GO. In addition to the ability of GO to generate ROS [14,42], ROS are also generated by the interaction of GO with mitochondria and chloroplasts [2,6]. After *Raphidocelis subcapitata* [43] and *Microcystis aeruginosa* [14,25] were exposed to GO for 96 h, it was found that GO concentrations above 10 mg/L significantly increased the intracellular ROS levels, damaging cell membranes, decreasing chlorophyll autofluorescence [14,25], and disrupting the chloroplasts ultrastructure [14,43] with the inevitable impact in the photosynthetic process. The most used method for evaluating membrane damage is the determination of lipid peroxidation level. Long et al. [38], Ouyang et al. [26], and Yin et al. [24] described increased or unaltered LPO levels in algae exposed to high GO concentrations. A rather opposite effect was observed in our study, with LPO levels being significantly lower at the higher GO concentrations (10 and 20 mg/L). However, this result should not lead us to conclude that in our study GO did not cause oxidative stress. Analyzing other biochemical markers, we can find that GO induced oxidative stress, as evidenced by increased protein damage, increased lipid content, increased carotenoid level, and augmented SOD and GST activity. SOD is considered the first line of defense against oxidative stress in cells. In our study, SOD activity was not significantly altered at low concentrations of GO, but at higher concentrations it increased significantly, evidencing the need to destroy the higher levels of ROS caused by high concentrations of GO. Yan et al. [24] also observed an increase in SOD activity for the five algae species studied, both for 1 and 10 mg/L GO, and Ouyang et al. [26] described an increase in SOD activity in *Chlorella vulgaris* exposed to GO concentrations between 0.01 and 10 mg/L. GSTs are also able to scavenge ROS and furthermore reduce toxic aldehydes resulting from lipid peroxidation, protecting cytosolic metabolism. Our results show that GST did not change at 0.1 mg GO/L, but significant increases in activity, that followed the increase in GO concentration, (1 to 20 mg/L) were observed. Algae accumulate lipids in stress situations [44]. Lipids accumulated by algae have a high degree of unsaturation [45]. In our study, lipid content did not change at 0.1 mg GO/L, but significantly increased for higher GO concentration, (1, 10, and 20 mg/L). These lipids can act as antioxidants by destroying ROS and decreasing oxidative stress. On the other hand, carotenoids that have antioxidant activity increased at high concentrations of GO and fucoxanthin (also a carotenoid abundant in diatoms) increased at all concentrations of GO. As part of the chloroplast membranes, carotenoids react with ROS, destroying them, thus preventing lipid peroxidation in the thylakoid membranes, and protecting the photosynthetic process. The increase in carbohydrates, which is an energy storage form for cells to quickly obtain energy, seems to indicate a reduction in the metabolic activity of cells, yet exposure to higher GO concentrations and the need to induce mechanisms protecting cells from higher levels of toxicity, gradually decreased carbohydrate levels, reaching at 20 mg/L of GO concentrations close to the control. The concerted action of all these mechanisms seems to destroy ROS so efficiently that the membrane integrity was preserved, and its level of peroxidation was reduced.

In this way, the third hypothesis, that GO can generate oxidative stress, causing oxidative damage, and inducing biochemical and physiological alterations, is partially confirmed. Actually, higher concentrations of GO induced oxidative stress and caused biochemical and physiological changes, such as an increase in lipids, carotenoids, and the activity of antioxidant enzymes. These mechanisms were so efficient that membranes were protected and protein oxidation was low.

## 4. Materials and Methods

### 4.1. Graphene Oxide Nanosheets

The commercial graphene oxide (GO) nanosheet water dispersion (0.4 wt% concentration) was purchased from Graphenea (San Sebastian, Spain) and was used as received. The GO nanosheets lateral size have a high variability (flakes mean size of ~790 nm and always <10 μm) and the monolayer a typical size of 0.97 nm, according to the supplier. We previously confirmed these data by atomic force microscopy (AFM) analysis with flakes having a typical sheet-like morphology, mostly (>95%) being monolayers together with few-layered nanosheets [46].

### 4.2. Biological Material and Experimental Setup

Periphyton was scraped from the Caima riverbed stones (epilithon) (40.696833 N, −8.462296 W) on December 2019 and transported in 50 mL falcons in ice to the lab. In the lab, 15 mL of biofilm were pipetted to plastic boxes paved with 15 ceramic tiles (4 × 5 cm), containing 500 mL of 10 times diluted Chu No.10 medium [47] and cultured under 10,100 lx in 12 h:12 h light/dark cycle at 18 ± 1 °C. After 24 h, graphene oxide (GO) at different concentrations (0, 0.1, 1.0, 10, and 20 mg/L) was added and the culture period extended for 96 h under the same growth conditions. The predicted environmental concentrations of this emerging contaminant in aquatic systems is projected to be approximately 0.001–1 mg/L [19,20,48]. Additionally, higher GO concentrations were used to ascertain the concentrations impacting the biofilm. Biofilm not exposed to GO (0 mg/L) was considered as the control condition. At the end of the growth period, tiles were scraped and the content centrifuged 10,000× *g* for 5 min at 4 °C. The supernatant was discarded, and the pellet used or frozen at −80 °C for later use.

### 4.3. SEM-EDX Analysis

From each condition, tiles with biofilms attached were used for Scanning Electron Microscopy (SEM). Tiles were freeze dried and the images obtained using a Hitachi TM4000 plus (Hitachi, Japan) using an accelerating voltage of 15 kV [49]. Energy-dispersive X-ray Spectroscopy (EDX) was performed to evaluate the chemical composition of biofilms exposed to the control (0 mg/L GO) and 20 mg/L GO. Samples were deposited on a glass plate and coated with carbon for SEM analysis. The optical spectra were recorded using a Jasco V-560 UV–vis spectrophotometer; for the solid samples, the spectra were recorded in the diffuse reflectance mode using MgO as the reference.

### 4.4. Photosynthetic Pigments

The fresh pellet was suspended in 90% acetone, protected from the light, and maintained in the cold. Samples on ice were sonicated for 20 s. The extract was centrifuged at 10,000× *g* for 10 min at 4 °C. Chlorophylls a, b, and c, carotenoids and fucoxanthin (a xanthophyll characteristic of brown algae and in freshwater communities present in diatoms) were determined spectrophotometrically, and the concentration calculated following the procedure of Nayek et al. [50] and Wang et al. [51]. Results were expressed relative to the control. Absolute values are available in the Supplementary Material (Table S1).

### 4.5. Exopolysaccharides (EPSs)

EPSs were extracted using the method described by Staats et al. [52] with some modifications [53]. Briefly, the fresh pellet was suspended in water at 55 °C for 30 min to remove cell wall associated polysaccharides. After centrifugation 8000× *g* for 2 min at 20 °C, the supernatant was collected and polysaccharide content immediately determined by the phenol–sulphuric acid method [54] using sucrose as a standard. To the supernatant, 20% phenol and 95% sulphuric acid were added in a 5:1:12 *v*/*v/v* ratio. After 30 min incubation, the mixture was pipetted to a microplate and the absorbance read at 490 nm. Results were expressed relative to the control. Absolute values are available in the Supplementary Material (Table S1).

### 4.6. Energy Related Parameters

Soluble sugars were extracted by suspension of frozen samples in 0.1 M potassium phosphate buffer (pH 7.4), sonication for 45 s at 50% amplitude with samples kept in ice to avoid heating, and centrifugation 10,000× *g* for 10 min at 4 °C. The supernatant was used to determine soluble sugars content using the same methodology used for EPS determination (Section 4.5). Results were expressed relative to the control. Absolute values are available in the Supplementary Material (Table S1).

Proteins (PROTs) were determined following the method described by Robinson and Hogden (1940) [55]. The same supernatant used for soluble sugars determination was used. The sample and Biuret reagent (1:10 *v*/*v*) were added to a microplate. After 10 min incubation in the dark, the absorbance was read at 540 nm and the concentration calculated using BSA as the standard. Results were expressed relative to the control. Absolute values are available in the Supplementary Material (Table S1).

Lipids (LIPs) were determined following the methodology by Folch et al. [56], modified by Cheng et al. [57]. Frozen pellets were dried at 50 °C overnight. A mixture of chloroform and methanol (2:1 *v*/*v*) was added to the dried pellet and incubated at 50 °C for 4 h. After incubation tubes were cooled in ice, 95% sulphuric acid was added, and samples were incubated for 10 min at 100 °C and then cooled in ice. To the cooled tubes, 0.2% vanillin (3-methoxy-4-hydroxybenzaldehyde) solution in 68.6% phosphoric acid (H3PO4) was added. The tubes were kept in the dark for 1 **h** and then samples were pipetted to a microplate and the absorbance was read at 520 nm. Cholesterol was used as the standard. Results were expressed relative to the control. Absolute values are available in the Supplementary Material (Table S1).

### 4.7. Antioxidant Enzyme Response

Superoxide dismutase (SOD) activity was measured following the Beauchamp and Fridovich (1971) methodology [58]. The same supernatant used for soluble sugars determination was used (Section 4.6). The sample, reaction buffer (50 mM Tris-HCl (pH 8.0), 0.1 mM DTPA, 0.1 mM Hypoxanthine) with tetrazolium salt (NBT), and xanthine oxidase (1:10:1 *v*/*v/v*) were added to a microplate and incubated for 20 min at room temperature with orbital rotation. Absorbance was measured at 560 nm. Blanks without NBT were also prepared. One unit of enzymatic activity (U) refers to a 50% inhibition of NBT reduction. Results were expressed relative to the control. Absolute values are available in the Supplementary Material (Table S1).

Glutathione S-transferase (GST) was determined following the method described by Habig et al. (1974) [59]. Sequentially, the sample and reaction solution (0.1 M potassium phosphate buffer, pH 6.5, 10 mM GSH, and 60 mM CNDB) (1:2 *v*/*v*) were pipetted to a microplate and the absorbance was read at 340 nm in 15 s intervals during 15 min. Results were expressed relative to the control. Absolute values are available in the Supplementary Material (Table S1).

### 4.8. Oxidative Damage

Lipid peroxidation (LPO) was measured by quantification of thiobarbituric acid reactive substances (TBARSs), according to the protocol described by Buege and Aust (1978) [60]. The frozen pellet was suspended in Trichloroacetic acid (TCA) (20%), and sonicated and centrifuged as in Section 2.4. Absorbance was measured at 535 nm (Ɛ = 1.56 × 10^5^ M^−^^1^·cm^−^^1^). Results were expressed relative to the control. Absolute values are available in the Supplementary Material (Table S1).

Protein carbonylation (PC) levels were determined following the alkaline method of Mesquita et al. [61] with some modifications [62]. The same supernatant used for soluble sugars determination was used (Section 4.6). The sample and 2,4-dinitrophenylhydrazine (DNPH) (1:1 *v*/*v*) were added to a microplate. After 10 min incubation, sodium hydroxide (NaOH) was added, and the reaction was incubated 10 more min. Absorbance was measured at 450 nm (Ɛ = 22,308 M^−^^1^·cm^−^^1^). Results were expressed relative to the control. Absolute values are available in the Supplementary Material (Table S1).

### 4.9. Statistical Analysis

All parameters assessed were submitted to hypothesis testing. Parameters were analysed following a one-way hierarchical design, with GO concentration (0, 0.1, 1, 10, and 20 mg GO7 L) as a fixed factor. The matrix resemblance was obtained using Euclidean distance. The effect of GO on samples was determined using pair-wise Permutational Multivariate Analysis of Variance (PERMANOVA) [63], using PRIMER version 6.1.16 for Windows [64].

The null hypothesis was: different GO concentrations (0, 0.1, 1.0, 10, and 20 mg/L) did not cause biochemical differences in biofilms. This hypothesis was tested for all parameters described. Values of *p* ≤ 0.05 revealed that conditions differed significantly, indicated in figures by different lowercase letters.

Matrix gathering of the biomarker responses (Chl a, Chl b, Chl c, Carot, Fuc, Lip, intra CH, LPO, prot, EPS, SOD, and GST) was used to calculate the Euclidean distance similarity matrix. The data used to calculate the matrix were previously normalized. The matrix was then simplified through the calculation of the distance among centroid based on the condition and then submitted to ordination analysis, performed by Principal Component Analysis (PCA). Based on the Pearson correlation vectors of biomarkers obtained (r > 0.50), it was possible to identify the biochemical parameters that imposed more differences when biofilms were exposed to GO.

## 5. Conclusions

The multiple uses of carbon-based nanoparticles implies they will be found in aquatic systems with consequences poorly known for aquatic life. In this study, we seek to respond to three effects of GO on organisms currently described in the literature: the mechanical effect, the shading effect, and the biochemical/physiological effect. The lower susceptibility to GO found in our study relative to other studies described in the literature may be due, not to the duration of exposure or the GO concentrations used, but to the biological model. In most studies, monoalgal cultures are used, while in our study the response of a biofilm is evaluated. These results are relevant and point to the importance of studying communities rather than individual organisms or species, since the results are markedly different and explain why it is often difficult to extrapolate to the environment results obtained in the laboratory. Biofilms, being complex entities, are more similar to environmental communities and may provide more accurate information to develop guidelines and legislation for the protection of aquatic environments. This study generated fundamental knowledge that may help to develop new tools based on cell responses at the biochemical, physiological, and metabolomics levels and to establish appropriate regulatory guidelines, allowing to predict and mitigate the impacts of mining activity in freshwater systems. Thus, results from this study can make a significant contribution to environmental risk assessment and environmental management strategies which will update relevant decision- and policymakers (both in the government and industry).

## Data Availability

All the data are embedded in the manuscript.

## Supplementary Materials

The following supporting information can be downloaded at: https://www.mdpi.com/article/10.3390/molecules28124577/s1, Table S1: Results obtained for biochemical parameters of biofilm exposed to different GO concentrations (0, 0.1, 1, 10, and 20 mg/L). Values are means of four replicates + standard deviation. Significant values were considered for *p* ≤ 0.05 and identified with letters; Table S2: Comparative table of the effects of GO published against our results. Alterations in photosynthetic pigments (chlorophyll a, b, and c, carotenoids and fucoxanthin), cell metabolism (lipid content and intracellular carbohydrates), antioxidant and biotransformation activity (SOD and GSTs), and cellular damage (reactive oxygen species, lipid peroxidation, and protein carbonylation).
