# Supplementary material for: Effects of Graphene Oxide Nanosheets in Freshwater Biofilms
Source: Molecules. 2023 Jun 6;28(12):4577. doi: 10.3390/molecules28124577 (PMC10304026; doi:10.3390/molecules28124577)
Supplement: Supplementary file 1 [file molecules-28-04577-s001.zip › Table S2.pdf]

Supplementary Table S2 – Comparative table of the effects of GO published against our results. Alterations in photosynthetic pigments (chlorophyll a, b and c, carotenoids and fucoxanthin), cell metabolism (lipid content and intracellular carbohydrates), antioxidant and biotransformation activity (SOD and GSTs) and cellular damage (reactive oxygen species, lipid peroxidation and protein carbonylation).

Parameter	Effect	Organism	[GO]	Reference
Chlorophyll a	Decrease	*Chlorella vulgaris, Scenedesmus obliquus*	10 mg/L	[24], [26]
	Increase	*Chlamydomonas reinhardti, Cyclotella* sp		[24]
	Decrease	*Mycrocystis aeruginosa*	20 mg/L	[25], [16]
	**Increase**	***Fluvial biofilm***	**10, 20 mg/L**	**Present study**
Chlorophyll b	**Increase**	**Fluvial biofilm**	**10, 20 mg/L**	**Present study**
Chlorophyll c	**Increase**	**Fluvial biofilm**	**10, 20 mg/L**	**Present study**
Carotenoids	**Increase**	**Fluvial biofilm**	**10 mg/L**	**Present study**
Fucoxanthin	**Increase**	**Fluvial biofilm**	**20 mg/L**	**Present study**
Lipid content	**Increase**	**Fluvial biofilm**	**10, 20 mg/L**	**Present study**
Intracellular carbohydrates	**Increase**	**Fluvial biofilm**	**0.1, 1, 10 mg/L**	**Present study**
SOD activity	Increase	*Chlorella vulgaris, Scenedesmus obliquus, Microcystis aeruginosa, Chlamydomonas reinhardtii, Cyclotella* sp	10 mg/L	[24]
	**Increase**	**Fluvial biofilm**	**10, 20 mg/L**	**Present study**
GSTs activity	**Increase**	**Fluvial biofilm**	**10, 20 mg/L**	**Present study**
ROS	Increase<br>Increase<br>Increase	*Chlorella vulgaris, Scenedesmus obliquus, Chlamydomonas reinhardtii, Cyclotella* sp	10 mg/L	[24]
	Increase	*Mycrocystis aeruginosa*	10, 20 mg/L	[24], [25]
Lipid peroxidation	Increase	*Chlorella sp.*	10, 20 mg/L	[38]
	**Decrease**	**Fluvial biofilm**	**10, 20 mg/L**	**Present study**
Protein carbonylation	**Increase**	**Fluvial biofilm**	**10 mg/L**	**Present study**
